# Impact of Resolvin-E1 and Maresin-1 on Bone Marrow Stem Cell Osteogenesis under Inflammatory Stress

**DOI:** 10.3390/cells13110932

**Published:** 2024-05-28

**Authors:** Shahd AlZahrani, Zakia Shinwari, Ayodele Alaiya, Ahmed Al-Kahtani

**Affiliations:** 1 Department of Restorative Dental Sciences, College of Dentistry, King Saud University, P.O. Box 60169, Riyadh 11612, Saudi Arabia; shahdsz93@gmail.com; 2 Therapeutics & Biomarker Discovery for Clinical Applications, Cell Therapy & Immunobiology Department, King Faisal Specialist Hospital and Research Centre, P.O. Box 3354, Riyadh 11211, Saudi Arabia; szakia@kfshrc.edu.sa (Z.S.); aalaiya@kfshrc.edu.sa (A.A.)

**Keywords:** bone regeneration, inflammation, mesenchymal stem cells, Maresin 1, osteogenesis, osteoclastogenesis, periodontal disease, Resolvin E1, specialized pro-resolving mediators

## Abstract

Periodontal disease is characterized by inflammation and bone loss. Central to its pathogenesis is the dysregulated inflammatory response, complicating regenerative therapies. Mesenchymal stem cells (MSCs) hold significant promise in tissue repair and regeneration. This study investigated the effects of specialized pro-resolving mediators (SPMs), Resolvin E1 (RvE1) and Maresin 1 (MaR1), on the osteogenic differentiation of human bone marrow-derived MSCs under inflammatory conditions. The stem cells were treated with SPMs in the presence of lipopolysaccharide (LPS) to simulate an inflammatory environment. Osteogenic differentiation was assessed through alkaline phosphatase activity and alizarin red staining. Proteomic analysis was conducted to characterize the protein expression profile changes, focusing on proteins related to osteogenesis and osteoclastogenesis. Treatment with RvE1 and MaR1, both individually and in combination, significantly enhanced calcified deposit formation. Proteomic analysis revealed the differential expression of proteins associated with osteogenesis and osteoclastogenesis, highlighting the modulatory impact of SPMs on bone metabolism. RvE1 and MaR1 promote osteogenic differentiation of hBMMSCs in an inflammatory environment, with their combined application yielding synergistic effects. This study provides insights into the therapeutic potential of SPMs in enhancing bone regeneration, suggesting a promising avenue for developing regenerative therapies for periodontal disease and other conditions characterized by inflammation-induced bone loss.

## 1. Introduction

Periodontal disease is a spectrum of inflammatory conditions affecting the supporting structures of the teeth. Characterized by gingival inflammation, periodontal ligament destruction, and alveolar bone loss, this disease can lead to tooth mobility and eventual loss if not adequately managed [1]. Apical periodontitis is a significant public health concern globally, affecting more than 1 billion people [2]. Central to the pathogenesis of periodontal disease is the role of inflammation initiated as a protective immune response to bacterial invasion. The inflammatory process can become dysregulated, leading to chronic inflammation and bone resorption [3]. The osteogenic potential of mesenchymal stem cells (MSCs), especially those derived from human bone marrow (hBMMSCs), offers a promising avenue for regenerating these lost tissues. MSCs can differentiate into osteoblasts under appropriate stimulatory conditions. Osteogenesis and osteoclastogenesis represent intricate processes governed by a network of cellular signals, hormones, growth factors, cytokines, and mechanical stimuli. Within this complex regulatory framework, inflammation and cytokines play a crucial role, exhibiting both beneficial and detrimental effects on bone resorption and remodeling [4,5]. However, the inflammatory microenvironment characteristic of periodontal disease can adversely affect the regenerative potential of hBMMSCs, challenging their therapeutic efficacy. Microbial factors, including endodontic microbiota and lipopolysaccharides (LPS), can exacerbate the inflammatory process, potentially impairing the bone regeneration capabilities of hBMMSCs [6,7].

Within this context, specialized pro-resolving mediators (SPMs), such as Resolvin E1 (RvE1) and Maresin 1 (MaR1), emerge as potential modulators of inflammation, with the added benefit of influencing bone metabolism [7]. SPMs are a group of bioactive lipid compounds derived from polyunsaturated fatty acids (PUFAs). They play a critical role in resolving inflammation without inducing immunosuppression [8]. Among SPMs, Resolvin E1 (RvE1) and Maresin 1 (MaR1) have garnered significant interest due to their potent anti-inflammatory and pro-resolving actions. These mediators not only suppress inflammation but also have been implicated in enhancing the regenerative processes in inflammatory conditions [9]. Distinct from conventional anti-inflammatory medications that primarily obstruct the onset of inflammation, SPMs actively facilitate the resolution process, ensuring the restoration of homeostasis while maintaining the integrity of host defense mechanisms [10].

SPMs function at the local level, regulating leukocyte recruitment and facilitating both the resolution of inflammation and subsequent bone healing [11]. These mediators operate through a variety of biosynthetic pathways, engaging specific cellular targets, and manifesting their effects in a temporally controlled fashion, which implies a tailored regulation for each SPM within this superfamily [12].

Previous studies have described the versatile roles of SPMs in modulating the behavior of various cell types involved in inflammatory response and tissue regeneration processes [13]. These investigations have provided valuable insights into the mechanisms by which SPMs, including Resolvin E1 (RvE1) and Maresin 1 (MaR1), influence immune cells, endothelial cells, and mesenchymal stem cells, facilitating the resolution of inflammation and promoting tissue healing and repair [14]. SPMs have been shown to regulate polymorphonuclear neutrophil (PMN) functions, such as reducing apoptosis and enhancing microbicidal activity, thereby supporting the clearance of inflammation and the maintenance of tissue homeostasis [15]. MSCs have been found to produce SPMs themselves, further underlining the autocrine and paracrine mechanisms through which SPMs can influence the local microenvironment to favor resolution and regeneration [16].

SPM-based treatments promote osteogenesis and bone remineralization. Improved osteogenesis has been associated with better periapical healing, reduced periapical radiolucency, and increased bone density in the periapical region [17]. On the other hand, inadequate bone healing or persistent periapical inflammation may impede the healing process and lead to treatment failure [18]. Systemic oral administration of pro-resolving mediators (omega-3 PUFAs) decreased the expression of the proinflammatory cytokines TNF-α, IL-1β, IL-6, and IL-17 and increased the production of the anti-inflammatory cytokine IL-10 in a rat model of apical periodontitis [6,19]. Systemic oral PUFA administration suppresses bone resorption and induces bone regeneration by decreasing osteoclastogenesis and increasing osteogenesis in apical periodontitis [20]. 

Resolvin E1 (RvE1) was reportedly instrumental in the regenerative process by moderating the initial inflammatory response in infected pulpal tissue, thereby facilitating the healing of both soft and hard dental tissues [21]. In an experimental model of immature rat teeth affected by apical periodontitis, the administration of RvE1 enhanced root development, apical closure, and tissue regeneration around the apex and suppressed the inflammatory responses [22]. When used as an intracanal dressing in immature rat molars RvE1 established a conducive biological environment for the differentiation of osteoblasts and cementoblasts, resulting in the formation of mineralized tissues and a reduction in periapical lesion size [22]. 

The application of MaR1 In the context of extraction socket wounds has shown promise in enhancing the healing process and improving the predictability of dental implant reconstruction [23]. MaR1 has been shown to express more osteoinductive properties by promoting the differentiation of bone marrow stem cells into osteoblasts in MaR1-treated bone marrow-derived macrophages [24]. Both MaR1 and RvE1 have been documented to revive the regenerative capacities of human periodontal ligament stem cells by enhancing cell viability, accelerating wound healing and cell migration, and upregulating markers indicative of periodontal ligament, cementogenic, and osteogenic differentiation [25]. RvE1 and MaR1 have been shown to restore regenerative capabilities by augmenting the viability of human periodontal ligament stem cells (hPDLSCs), accelerating wound healing and cell migration, and upregulating markers of periodontal ligament and cementogenic-osteogenic differentiation [25,26]. 

These findings highlight the potential of SPMs to not only facilitate healing in the dental and periodontal context but also to regulate the differentiation pathways critical for the regeneration of dental tissues. Given this background, this study aimed to specifically investigate the effects of RvE1 and MaR1, both individually and in combination, on the osteogenic differentiation of hBMMSCs within an inflammatory setting.

## 2. Materials and Methods

### 2.1. Cell Culture and Characterization

Human bone marrow-derived mesenchymal stem cells (hBMMSCs), confirmed to be free of mycoplasma contamination and immortalized, were generously provided by the Stem Cell Laboratory at King Saud University. These cells were cultured in DMEM-high glucose medium (Sigma-Aldrich, St. Louis, MO, USA) supplemented with 10% FBS (Gibco, Carlsbad, CA, USA), 4 mM L-glutamine (Gibco, Carlsbad, CA, USA), and 1 mM sodium pyruvate (Sigma-Aldrich, St. Louis, MO, USA) and incubated at 37 °C with 5% CO_2_. Characterization of these cells has been detailed in our previous paper [5]. 

### 2.2. Drug Preparation and Optimization

Synthetic specialized pro-resolving inflammatory mediators RvE1 and MaR1 were purchased commercially (GLPBIO Technology, Montclair, CA, USA). Stock solutions (10 µM) of each were prepared in absolute ethanol and stored at −80 °C for further use. To optimize the concentration of different treatments of RvE1 and Mar1, hBMMSCs were exposed to 10, 50, 100, and 150 nM of RvE1, MaR1, or control (medium), then the proliferation rate was determined using a label-free real-time cell analysis platform (xCELLigence) (ACEA Biosciences, Santa Clara, CA, USA) with a cell load of 5K. An annexin V-FITC/PI double staining assay was used to assess the induced cellular death rate of hBMMSCs after RvE1 and MaR1 treatment, as previously described [5]. The doses were based on previous dose–response studies [26,27] and our experimental laboratory investigations. After comprehensively analyzing the results, the 100 nM concentration of RvE1 and MaR1 was selected for further experiments.

### 2.3. Experimental Design

hBMMSCs were seeded into two sets of three 6-well plates, and coated with 10 µg/mL fibronectin (to enhance cell adhesion, growth, and differentiation) at a density of 8 × 10^5^/well. Upon confluence (100%), the wells were arranged into two sets of five treatment groups: the first set had an incubation period of 7 days while the second had 14 days. The treatment groups were designed as follows: (1) Negative control (unstimulated/no LPS), (2) Positive control (1 μg/mL LPS stimulated), (3) RvE1 100 nM + 1 μg/mL LPS, (4) MaR1 100 nM + 1 μg/mL LPS, and (5) RvE1 100 nM + MaR1 100 nM + 1 μg/mL LPS. The medium of the confluent cells was replaced with an osteogenic differentiation medium supplemented with the proper dose of LPS, RvE1, and MaR1. For each experiment, the same passage of cells was used. Cells were incubated at 37 °C in 5% CO_2_, and the results will be confirmed by making at least three measurements.

### 2.4. Osteogenic Differentiation of Treated hBMMSCs

Alkaline phosphatase (ALP): After 14 days, an ALP activity assay was conducted to assess osteoblastic differentiation. hBMMSCs were cultured in coated 12-well plates with 10 µg/mL fibronectin at a density of 2 × 10^4^/well. Upon confluence, the cells were treated and differentiated using an osteogenic differentiation medium as mentioned earlier. Cells were then washed with Ca++/Mg++-free PBS and fixed using 10% neutral buffer formalin (Sigma Aldrich, Munich, Germany). Subsequently, the plate was incubated at room temperature for 60 to 90 s. Following this, cells were washed with wash buffer (Ca++/Mg++-free PBS plus 0.05% Tween 20). Substrate solution 5-Bromo-4-chloro-3-indolyl phosphate/Nitro blue tetrazolium (BCIP/NBT) was freshly prepared by dissolving one tablet in 10 mL distilled water. Cells were coated with prepared BCIP/NBT and kept in the dark for 10 min before removing the substrate and washing the cells with washing buffer and then PBS. Differentiated cells stained blue-violet in the presence of ALP.

Alizarin red staining: to verify the calcium deposition, cells were cultured in 12-well plates coated with 10 µg/mL fibronectin at a density of 2 × 10^4^/well. Upon confluence, the cells were treated and differentiated using an osteogenic differentiation medium as mentioned earlier at the same time point (day 14). Cells were then washed with Ca++/Mg++-free PBS and fixed with 10% neutral buffer formalin for 30 min (Sigma Aldrich, Munich, Germany). Subsequently, cells were coated with alizarin red S solution (pH 4.2; ScienCell™, Carlsbad, CA, USA) and kept in the dark for 15 min before removing the dye and washing the cells four times with double distilled water and finally PBS. Undifferentiated cells were colorless or slightly purple, whereas for MSC differentiated osteogenic cells were bright orange-red.

All samples were observed, and digital images were obtained using a digital microscope (Leica Microsystems, Wetzlar, Germany) at various magnifications. Mineral deposits were quantified by calculating the total optical density using The PDQuest^TM^ Software (v7.3.1; Bio-Rad Laboratories, Hercules, CA, USA). To ensure the reproducibility and reliability of our findings, all experiments were independently replicated twice.

### 2.5. Enzymatic Protein Digestion and Preparation of Peptides before MS Analysis

Before liquid chromatography mass spectrometry analysis, complex protein mixtures in samples were subjected to in-solution enzymatic protein digestion by trypsin, as previously described [5]. Briefly, hBMMSCs from the three sample groups were lysed in RapiGest SF buffer (Waters, Manchester, UK). Because of the inherent low throughput of LC/MS/MS-based quantitative analysis, we pooled equal amounts (100 μg) of the total complex protein mixtures from each of the treated and untreated sample groups for proteomics analysis. The pools of each sample group were denatured at 80 °C for 15 min. Reduction of disulfide bonds was achieved by the addition of 10 mM DTT at 60 °C (30 min) and then alkylated in the dark at room temperature in 50 mM Iodoacetamide for 40 min. Sequencing grade trypsin (Promega, WI, USA) was added at a ratio of 1:50 (*w*/*w*) to all samples and left overnight at 37 °C with mild agitation. The tryptic digestion reaction was ceased with 37% hydrochloric acid, and the clear supernatant was carefully removed following centrifugation at 13,000 rpm for 10 min at 8 °C. Before liquid chromatography/mass spectrometry analysis, the samples were diluted in 0.1% formic acid and spiked with a known protein: alcohol dehydrogenase (ADH, P00330) for absolute quantification.

### 2.6. Protein Characterization by Label-Free Liquid Chromatography/Mass Spectrometry

Protein characterization of all samples was conducted using a Synapt G2 HD Mass Spectrometry instrument, coupled with a one dimensional (1D) nanoACQUITY liquid chromatography system (Waters, Manchester, UK). The instrument calibration and optimization settings were carried out using the MassLynx tune page prior to analysis, as previously described [5]. Briefly, 2 ng/L of leucine enkephalin and 500 fmol [Glu]1-fibrinopeptide B were used by the Mass Lynx IntelliStart for detector setup and mass calibration, respectively. Other parameters included a capillary voltage of 3 kV, 50 and 5 V for the sample cones and extraction cones, respectively, and a source temperature of 85 °C. All samples were run in Trizaic Nano Source, in the positive ion mode nanoESI. Equal amounts of protein digest (3 μg) per sample were loaded, trapped, and exchanged in the AcquityTM HSS T3 85 µm × 100 mm Trizaic Nano tile column (Waters, Manchester, UK). A flow rate of 0.450 μL/min was used in the mobile phases of A1 (99% water, 1% Acetonitrile + 0.1% formic acid) and B1 (100% Acetonitrile + 0.1% formic acid). We used data-independent acquisition (DIA) (MSE)/ion-mobility separation experiments and acquired data over a range of *m*/*z* 50–2000 Da with a scan time of 1 s, and collision energy 20–50 V with a gradient run of a total acquisition time of 2 h, as previously described [5]. We analyzed all samples in duplicate runs and all data acquired using Mass Lynx (version. 4.1, SCN833; Waters, Manchester, UK), which was operated in resolution and positive polarity modes. 

### 2.7. Data Analysis

The acquired raw MS data were processed and database searching was accomplished using the Progenesis LC-MS proteomics data analysis software (Progenesis qIfP V4.0 (Waters, Manchester/Nonlinear, Newcastle, UK) as previously reported [28]. The acquired list of peptide ions was queried using the non-redundant UniProt/SwissProt human-specific (Homo sapiens) protein sequence database for protein identification (www.uniprot.org, accessed on 12 June 2023). The generated data were filtered using multivariate statistical analysis and differential expression analysis. Only proteins with expression changes with a *p*-value < 0.05 and at least ≥2-fold were considered statistically significant. We made measures to overcome the inherent problems of multiple testing leading to a False Discovery Rate (FDR). Therefore, the applied adjusted *p*-value or *q*-value was calculated by the embedded algorithm in the licensed Progenesis qIfP program. In addition, power thresholds as well as a minimum of 1.5-fold change were considered in the criteria to define our differentially expressed protein. We applied multivariate data analysis to identify only statistically significant regulated proteins that were showing greater than 1.5-fold abundance change and a *p*-value < 0.05 between pairs of samples being compared using the tools embedded in Progenesis QI.

### 2.8. Statistical Analysis

The data from the ALP and alizarin red quantification assays were expressed as the mean of three experiments ± the standard deviation (SD), and were analyzed using one-way analysis of variance (ANOVA) followed by Bonferroni’s multiple comparison post-test using GraphPad Prism^®^ (GraphPad Software, Inc., Boston, MA, USA) statistical software version 5.01. Statistical differences yielding *p* < 0.05 were considered significant.

## 3. Results

### 3.1. Osteogenic Differentiation

**Figure 1:**
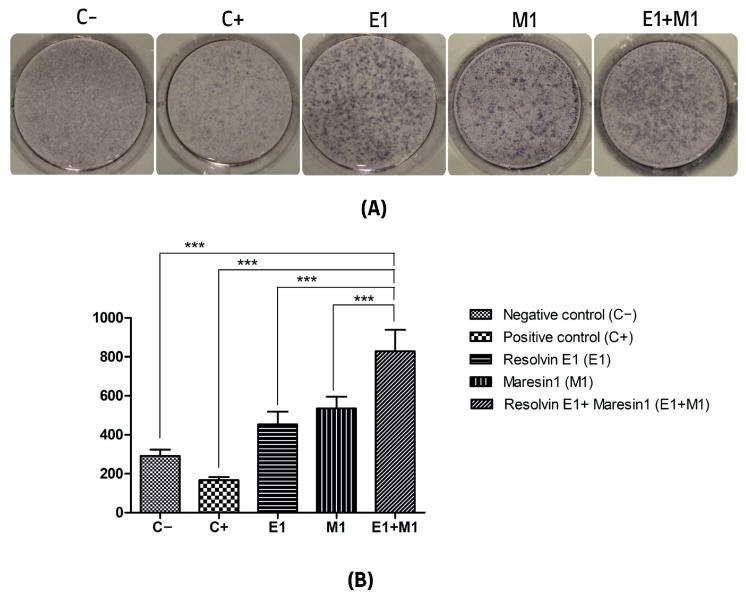
(**A**) Alkaline phosphatase (ALP) activity. (**B**) Quantitative analysis of the average intensity of each well of mineral nodule formation. The y-axis shows the quantitation as a measure of the optical density of the scanned wells. (C−, Negative control cell; C+, positive control cells; E1, Resolvin E1-treated cells; M1, Maresin 1-treated cells; E1+M1 Combined Resolvin E1- and Maresin 1-treated cells). (*** *p* ≤ 0.0001).

**Figure 2:**
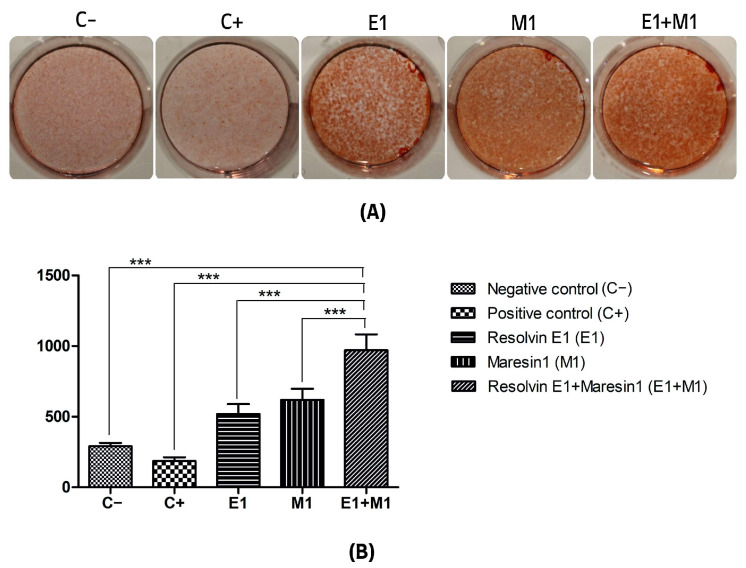
(**A**) Alizarin red staining mineralization assay of calcium deposition. (**B**) Quantification of staining intensity represented as an average of optical density of staining intensity of the scanned wells. (C−, Negative control cell; C+, positive control cells; E1, Resolvin E1-treated cells; M1, Maresin 1-treated cells; E1+M1 Combined Resolvin E1- and Maresin 1-treated cells). (*** *p* ≤ 0.0001).

The analysis comparing the mean values of mineral deposit production over the study period, using alkaline phosphatase (ALP) activity and alizarin red staining as indicators, revealed a significant increase in mineral nodule formation in samples treated with specialized pro-resolving mediators (SPMs) compared to the control. Specifically, the group exposed to 1 μg/mL lipopolysaccharide (LPS) served as a positive control and showed the lowest mineralization levels, highlighting the negative impact of pro-inflammatory conditions on the osteogenic capabilities of mesenchymal stem cells (MSCs). Treatment with Maresin 1 (MaR1) and Resolvin E1 (RvE1) individually led to enhanced accumulation of calcified deposits and ALP activity. The cohort receiving the combined treatment of RvE1 (100 nM) and MaR1 (100 nM) along with 1 μg/mL LPS displayed the most pronounced increase in total optical density measurements among all groups evaluated, with a statistical significance of *p* < 0.0001. This enhancement was quantitatively assessed by calculating the mean intensity from each of the five wells to construct the corresponding histograms shown in Figure 1 and Figure 2.

### 3.2. Proteomics Enrichment Analysis and Characterization of Osteo-Related Proteins

Whole-cell lysates from human bone marrow-derived mesenchymal stem cells (hBMMSCs), spanning three experimental groups (E1, M1, and E1+M1) and two distinct treatment durations (7 and 14 days), underwent untargeted label-free quantitative proteomic analysis. This methodological approach aligns with the procedures detailed in our preceding publication [5]. In total, our analysis revealed the identification of 1975 and 1703 unique protein species across all samples for the 7-day and 14-day treatment periods, respectively. A significantly higher quantity of proteins, numbering 1028, exhibited changes in expression greater than a 2-fold increase with a *p*-value of ≤0.05 after 7 days of treatment. In contrast, the 14-day treatment period saw only 438 proteins demonstrating such significant changes in expression, as shown in Table 1. Comprehensive details concerning these proteins, including their accession numbers, identified peptides, *p*-values, fold changes, and descriptions, are provided in Supplementary Tables S1 and S2.

### 3.3. Protein–Protein Interactions and Annotations of Protein Expression Changes in 7 Days Treatment

**Figure 3:**
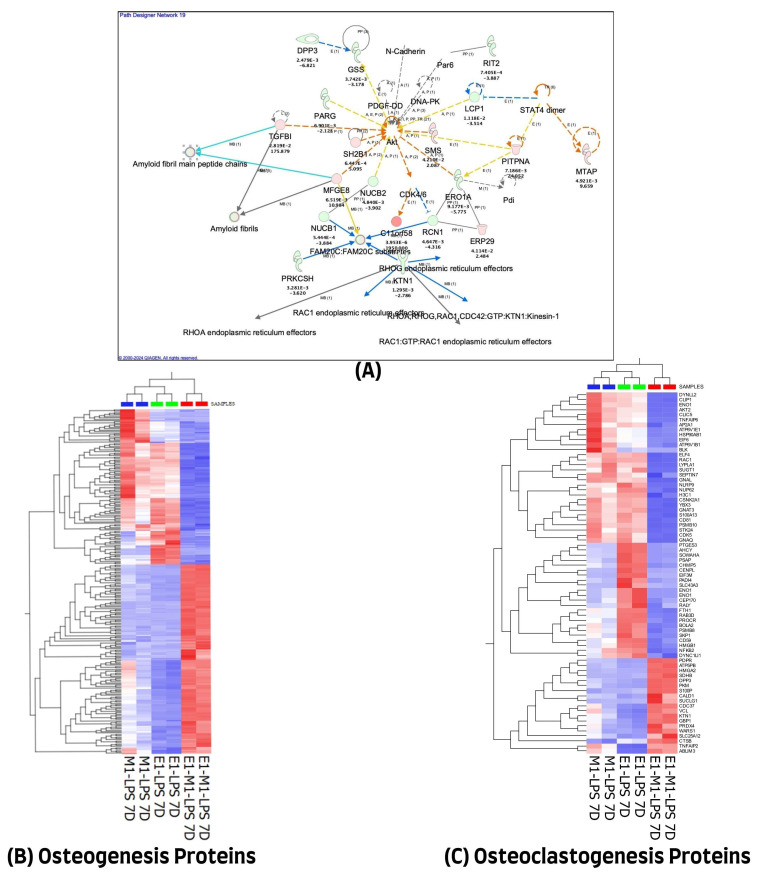
(**A**) Ingenuity Pathway Analysis (IPA) of 1028 differential proteins linked with osteogenic response on day 7. (The figure was partly generated using the licensed Ingenuity Pathway Analysis program (Version, 01-22-01, accessed on 20 July 2023) (www.qiagen.com)). (**B**) Hierarchical cluster analysis using the expression dataset of the 255 osteogenesis-related proteins. (**C**) Hierarchical cluster analysis using the expression dataset of the 70 osteoclastogenesis-related proteins. The heat maps show the expression changes of these proteins among the three treated groups. The images were generated using Qlucore Omics Explorer version 3.7, accessed on 1 August 2023 (Lund, Sweden, https://qlucore.com/).

We used the QIAGEN knowledge-based Ingenuity Pathway Analysis program (IPA, Qiagen, Germantown, MD, USA) to further evaluate the 1028 differentially expressed proteins in the 7-day treatment group for their protein–protein interactions and their molecular functional annotations. A total of 971 of the 1028 differentially expressed proteins (DEP) were mapped in the IPA database, of which 474 were up-regulated and 497 were down-regulated in combined E1+M1, compared with single treatment agents. These proteins were implicated in multiple networks that are of relevance to this study. Among the interesting networks are developmental disorders, hereditary disorders, and RNA post-transcriptional modification, cell death and survival, hematological and immunological diseases. Others are connective tissue disorders, free radical scavenging, nucleic acid metabolism, and small molecule biochemistry. The protein–protein interactions of some of these proteins showing direct or indirect connections with some of the known inflammatory and osteogenesis pathways are illustrated in Figure 3A. The list of molecules and their cellular localizations and functions are detailed in Supplementary Table S3. A review of the literature identified 255 of the 1028 proteins as being associated with the osteogenesis process. However, only 70 of the 1028 differentially expressed proteins were implicated in the osteoclastogenesis process. The expression changes of these differentially expressed proteins, using unsupervised hierarchical cluster analysis with the heat map depicting the differences in their protein expression profiles of osteogenesis and osteoclastogenesis between the three treated sample groups, are illustrated in Figure 3B,C.

### 3.4. Protein–Protein Interactions and Annotations of Protein Expression Changes in 14 Days Treatment

A similar analytical approach, as described above, was applied to cells treated for 14 days. The majority (393 of the 438 differentially expressed proteins) were mapped in the Ingenuity Pathway Analysis (IPA). Unlike in the 7-day treatment, 287 of the 393 proteins were down-regulated, and only 106 proteins were up-regulated in the combined E1 + M1 group compared with the single-agent treatment. Similarly, these proteins were implicated in multiple networks that are of interest to this study. Among the interesting networks are cell morphology, post-translational modification, protein folding, cell-mediated immune response, and cellular development, function, and maintenance. Others include cancer, hematological and immunological diseases, cellular assembly and organization, cellular movement, organismal injury, and abnormalities.

**Figure 4:**
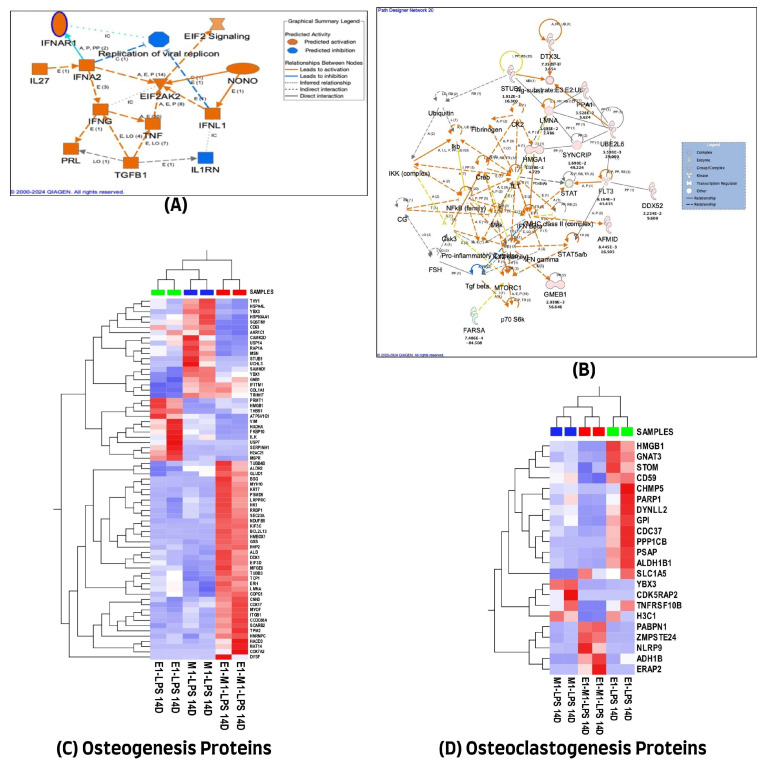
(**A**) Graphical summary illustrating the 14-day treatment molecules actions across all experimental groups. (**B**) Ingenuity Pathway Analysis (IPA) of 438 differential proteins linked with osteogenic response on day 14. (The figure was partly generated using the licensed Ingenuity Pathway Analysis program (Version, 01-22-01, accessed on 20 July 2023) (www.qiagen.com)). (**C**) Hierarchical cluster analysis using the expression dataset of the 69 osteogenesis-related proteins. (**D**) Hierarchical cluster analysis using the expression dataset of the 22 osteoclastogenesis-related proteins. The heat maps show the expression changes of these proteins among the three treated groups. The images were generated using Qlucore Omics Explorer version 3.7, accessed on 1 August 2023 (Lund, Sweden, (https://qlucore.com/).

Protein–protein interaction analysis revealed both direct and indirect connections of several proteins with established pathways involved in inflammation and osteogenesis, as depicted in Figure 4B. These interactions varied, with some proteins promoting activation while others had inhibitory effects, as detailed in the graphical summary presented in Figure 4A. A comprehensive list of these proteins, along with their cellular localizations and functions, is provided in Supplementary Table S4.

Additionally, a literature review determined that out of 438 proteins exhibiting differential expression, only 69 were associated with the osteogenesis process. However, a smaller subset of 22 proteins (out of the same 438) was found to play a role in osteoclastogenesis. The variations in protein expression related to osteogenesis and osteoclastogenesis across the three experimental groups were visualized through unsupervised hierarchical cluster analysis, with the resulting heat maps displayed in Figure 4C,D, highlighting the differential protein expression profiles.

### 3.5. Overlapped Proteins Observed at Both 7 and 14 Days

**Figure 5:**
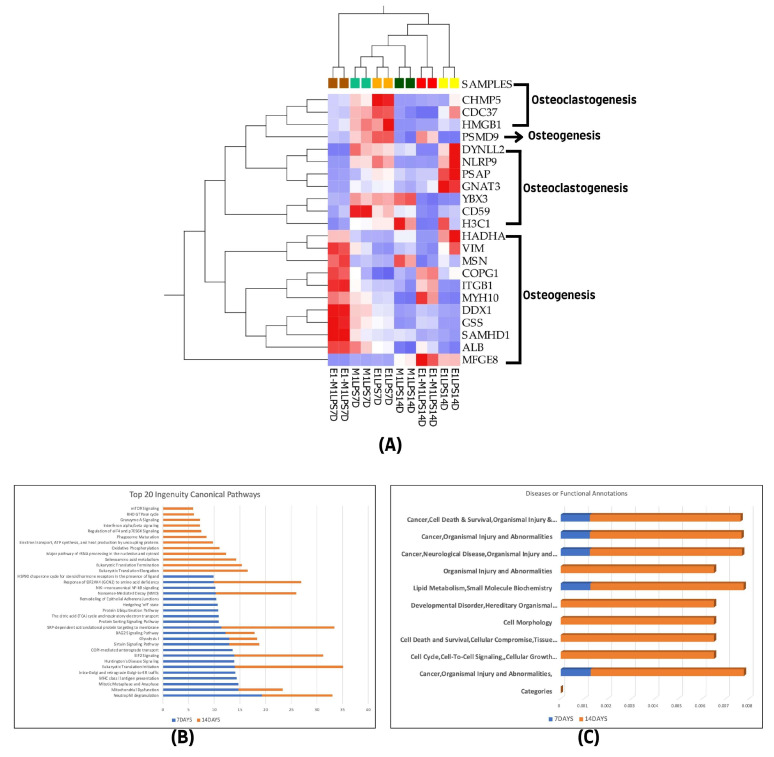
(**A**) Hierarchical cluster analysis using the expression data set of the 22 overlapped differentially expressed proteins among the experimental groups of the two treatment time points (7- and 14-day treatment groups). (**B**) The graphical canonical pathway representations using ranked log *p* values, and (**C**) disease or functional annotation representations for 7- and 14-day treatment groups. The graphs were derived using quantitative information from IPA analysis of the 1028 and 438 differentially expressed proteins of the 7- and 14-day treatment groups.

We determined the relatedness between the differentially expressed proteins at the two evaluated treatment intervals. A notable overlap of 22 proteins associated with both osteogenesis and osteoclastogenesis was identified at the 7-day and 14-day marks. Specifically, from the 255 proteins linked to osteogenesis identified on day 7 and the 69 on day 14, a core set of 12 proteins was consistently observed across all samples, including ALB, ITGB1, VIM, MSN, MYH10, HADHA, GSS, COPG1, SAMHD1, DDX1, PSMD9, and MFGE8. In contrast, for osteoclastogenesis, 70 proteins were identified at the initial 7-day interval and 22 at the 14-day interval, with a subset of 10 proteins, namely CDC37, GNAT3, PSAP, HMGB1, CD59, YBX3, H3C1, NLRP9, CHMP5, and DYNLL2, consistently present at both time points. This consistency is visually represented in the heatmap in Figure 5A.

Further Ingenuity Pathway Analysis (IPA) was conducted on the top canonical pathways and diseases, as well as functional annotations for each treatment group, using ranked transformed log *p* values for comparison. This analysis revealed that both canonical pathways and disease and function categories showed a higher representation in the 14-day treatment group compared to the 7-day group, as shown in Figure 5B,C.

## 4. Discussion

The development and progression of the inflammatory process is closely connected and regulated by the action of the immune system, leading to the process of immunomodulation. This process can be influenced by a number of agents, such as the pharmacological and functional properties of some specialized cells. We investigated the effects of specialized pro-resolving mediators (SPMs), Resolvin E1 (RvE1) and Maresin 1 (MaR1), on the osteogenic differentiation capabilities of human bone marrow-derived mesenchymal stem cells (hBMMSCs) within an inflammatory microenvironment. 

The results unequivocally demonstrate a substantial enhancement in mineral deposit formation and osteogenic differentiation among hBMMSCs subjected to SPM treatments, compared to control groups. Notably, the combined application of RvE1 and MaR1 was distinguished by its superior efficacy in promoting calcified deposit accumulation. This synergistic effect was most pronounced in the presence of a pro-inflammatory stimulus, lipopolysaccharide (LPS), where the treatment group receiving both RvE1 and MaR1 exhibited significantly higher total optical density values of mineralization, compared to other experimental groups. 

The presence of LPS, a potent inducer of inflammation, typically exacerbates the pathological environment, leading to reduced osteogenic activity of mesenchymal stem cells (MSCs) [29]. This reduction in osteogenic activity can significantly impair bone repair and regeneration, a critical concern in conditions such as periodontal disease, where inflammation-driven bone loss is a hallmark. Our finding proved that the application of SPMs, specifically MaR1 and RvE1, enhances the osteogenic differentiation of hBMMSCs, as evidenced by increased mineral deposition. This is noteworthy given the challenging pro-inflammatory conditions imposed by LPS treatment. This suggests that SPMs can effectively counteract inflammatory signals that otherwise suppress bone formation processes.

The mechanism by which SPMs promote calcified deposit formation in the face of inflammation likely involves the modulation of the inflammatory response, promoting the resolution of inflammation while simultaneously enhancing the regenerative capacity of MSCs [30,31]. SPMs are known for their dual roles in actively resolving inflammation and promoting tissue repair, making them uniquely suited to address the complex interplay between inflammation and regeneration [32]. By dampening the pro-inflammatory setting and fostering a pro-regenerative environment, SPMs facilitate the osteogenic potential of MSCs even under adverse conditions [30]. This finding has significant implications for the development of therapeutic strategies aimed at bone regeneration, particularly in conditions characterized by chronic inflammation, such as periodontal disease, osteoporosis, and arthritis [33].

RvE1 has been recognized for its role in bone remodeling, primarily by inhibiting osteoclast differentiation. This effect is mediated through the modulated activity of NFκB and the phosphatidylinositol 3-kinase-protein kinase B (PI3K–AKT) signaling pathways, alongside maintaining levels of osteoprotegerin (OPG) that are otherwise diminished by proinflammatory mediators [13,34]. Consequently, RvE1 contributes to a decreased RANKL/OPG ratio, favoring bone formation [30]. Moreover, Maresin 1 has demonstrated the ability to reduce proinflammatory cytokines and ameliorate pathohistological changes which support tissue regeneration and inflammatory resolution by restoring mesenchymal stem cell function, highlighting its potential for organ regeneration, tissue repair, and modulation of stem cell activities [35]

Similarly, in our previous research, MSCs stimulated with lipopolysaccharide (LPS) exhibited a decrease in proinflammatory cytokines (TNF-α, IFN-γ, and RANKL) and an increase in anti-inflammatory cytokines (TGF-β, IL-10, and IL-4) following treatment with RvE1 and MaR1 [5]. A combined application of these agents notably enhanced the anti-inflammatory microenvironment, effectively reducing pro-inflammatory responses [5].

Recent research highlights the role of MaR1 and RvE1 in augmenting the regenerative functions of periodontal ligament stem cells within inflammatory contexts [25]. RvE1 has been shown to promote Lipoxin A4 production, which subsequently induces interleukin-10 (IL-10) synthesis in the presence of annexin-1 [31,32]. Our study corroborates these findings, with MSCs treated with a single specialized pro-resolving mediator exhibiting distinct differentiation patterns compared to untreated control groups [33]. This further substantiates the therapeutic potential of SPMs in tissue regeneration.

To further explore the global signaling changes induced by treatment with these specialized pro-resolving lipid mediators, quantitative proteomics analysis was conducted. This analysis identified a pronounced overexpression of proteins associated with osteogenesis and, to a lesser extent, proteins linked to osteoclastogenesis. These proteins play critical roles in pathways that are crucial for bone regeneration. Remarkably, the combined treatment group (RvE1 + MaR1) exhibited a more substantial increase in the expression of osteogenesis-related proteins, compared to the group treated with RvE1 or MaR1 alone. Our findings are consistent with previous research that showed RvE1 could enhance root development, apical closure, and tissue regeneration near the apex, while concurrently suppressing inflammatory responses in cases of apical periodontitis in animal models [22]. Similarly, MaR1 has been shown to decrease proinflammatory cytokine expression, primarily through inhibiting the NF-κB pathway and promoting the activation of M2 macrophages. This modulation not only influences pain mechanisms but also supports tissue regeneration [34]. Beyond its impact on proinflammatory cytokines, MaR1 exhibits osteo-inductive effects by enhancing the differentiation of bone marrow stem cells into osteoblasts in environments treated with MaR1 [24]. The interplay between these specialized pro-resolving mediators, where one mediator can trigger the production of another, establishes positive feedback mechanisms crucial for effectively resolving inflammation.

Our proteomic analysis revealed a temporal variation in the protein expression profile of hBMMSCs following SPM treatment. We identified 1975 and 1703 unique protein entities at 7- and 14-day intervals, respectively, revealing a dynamic proteomic landscape influenced by SPM treatment. Notably, a substantial increase in significant protein expression changes was observed at the 7-day interval. This pronounced response contrasts with the 14-day interval, where fewer proteins exhibited significant changes, indicating a temporal specificity in the proteomic response to SPM treatment. This highlights the potent and immediate impact of SPMs on the proteomic expression of hBMMSCs, suggesting a rapid initiation of signaling pathways conducive to osteogenesis and inflammation resolution. The temporal decline in the number of significantly altered proteins from 7 to 14 days may reflect a transition from active cellular response to a more stabilized state conducive to differentiation and tissue regeneration. This temporal pattern of protein expression changes underscores the critical window of therapeutic intervention following SPM administration for maximizing bone regeneration and healing processes.

Our results specifically point to the mineralization process, where a 14-day SPM treatment regimen shows more pronounced effects than a 7-day regimen. This distinction emphasizes the critical role of extended SPM exposure in altering the inflammatory microenvironment and fostering osteogenic differentiation in hBMMSCs. The superior results from the 14-day regimen suggest that a longer treatment period may be necessary for effectively resolving inflammation and augmenting the osteogenic capacity of hBMMSCs, leading to improved therapeutic effectiveness. Therefore, these findings underline the clinical implications of extending the duration of SPM treatment for managing LPS-induced inflammation and related bone disorders.

The observation of 22 overlapping proteins across the two examined time points of 7 and 14 days within the proteomics enrichment analysis provides significant insight into the persistent molecular mechanisms influenced by treatment with SPMs in the context of osteogenesis and osteoclastogenesis. Among these, a consistent set of 12 proteins associated with osteogenesis were identified across all samples, including ALB [35], ITGB1 [36], VIM [37], MSN [38], MYH10 [39], HADHA [40], GSS [41], COPG1 [42], SAMHD1 [43], DDX1 [44], PSMD9 [45], and MFGE8 [46]. Conversely, a subset of 10 proteins consistently implicated in osteoclastogenesis at both time points were identified, namely CDC37 [47], GNAT3 [48], PSAP [49], HMGB1 [50], CD59 [51], YBX3 [52] H3C1 [53], NLRP9 [54], CHMP5 [55], and DYNLL2 [56]. 

The consistent expression of these proteins across both treatment intervals underscores their pivotal roles in the regulation of bone metabolism processes. These proteins likely represent key components of the signaling networks that mediate the balance between bone formation and resorption, a balance that is crucial for the maintenance of skeletal integrity and the repair of bone tissue. The presence of these proteins at both 7 and 14 days suggests that the effects of SPM treatments on hBMMSCs have both immediate and sustained impacts on the cellular machinery governing osteogenic and osteoclastogenic activities.

The identification of these overlapping proteins not only enriches our understanding of the molecular basis of SPM-mediated enhancement of osteogenic differentiation and inflammation resolution but also provides potential targets for therapeutic intervention. By focusing on these consistently expressed proteins, future research could elucidate more precise mechanisms by which SPMs influence bone regeneration and identify novel strategies for enhancing bone healing in pathological conditions characterized by imbalanced bone remodeling, such as osteoporosis, arthritis, and periodontitis. The characterization of these proteins offers a unique opportunity to explore the duality of the effects of SPMs on both promoting bone formation and limiting excessive bone resorption. This dual action is particularly relevant in the context of periodontal disease, where inflammation-driven bone loss is a major concern.

Our study highlights the role of specialized pro-resolving mediators in osteogenesis and protein expression in human bone marrow-derived mesenchymal stem cells but also underscores inherent limitations. The conducted in vitro study’s conditions do not mirror the in vivo environment’s complexity, where factors like cellular interactions and mechanical forces play a critical role in bone regeneration and inflammation resolution. Furthermore, using lipopolysaccharide to simulate inflammation may not fully represent the diverse inflammatory landscape of diseases like periodontitis, osteoporosis, or arthritis, potentially limiting the applicability of our findings. Studies have proven that Western blotting data are commonly used as a symbolic representation of a validation method for quantitative proteomics data when faced with reliability limitations [57,58]. Antibody specificity is a major bottleneck and more importantly, the lack of commercially available antibodies to a specific panel of novel identified proteins of interest precludes the use of Western blot as a validation method. We have used global proteomics analysis as an unbiased method to separate complex protein mixtures and unravel proteins that are specific or related to changes induced by the different SPMs, based on reviewed canonical pathways in IPA, as well as a review of the literature of published data to confirm the relatedness of the identified protein panels to the treatment effects of SPMs. This approach offers an indirect and a better validation method of our findings, as reported in this study.

Future studies should aim to validate the osteogenic potential of SPMs in in vivo models of bone regeneration and periodontal disease to help understand the complex interactions between SPMs, inflammatory microenvironments, and bone tissue regeneration in a living organism, providing insights into the therapeutic efficacy of SPMs in clinical settings.

## 5. Conclusions

Based on our findings, Resolvin E1 and Maresin 1 promoted osteogenic differentiation of human bone marrow-derived mesenchymal stem cells in an inflammatory environment, with their combined application yielding synergistic effects. Our findings provide insights into the therapeutic potential of SPMs in enhancing bone regeneration, suggesting a promising avenue for developing regenerative therapies for periodontal disease and other conditions characterized by inflammation-induced bone loss. Further research is needed to elucidate the precise mechanisms through which SPMs exert their regenerative effects and to explore their clinical applications in periodontal regeneration.

## Figures and Tables

**Table 1:** Overview of the differentially expressed osteogenic-related proteins observed in both 7-day and 14-day treatment durations, categorized by the treatment groups and the number of highly expressed proteins.

Treatment Duration	Process	Total Proteins Identified	RvE1 + MaR1	MaR1	RvE1
7 days	Global total	1975			
	Differentially expressed	1028			
	Osteogenesis	255	139	64	52
	Osteoclastogenesis	70	19	20	31
14 days	Global total	1703			
	Differentially expressed	438			
	Osteogenesis	69	38	17	14
	Osteoclastogenesis	22	4	5	13
	Total identified osteo-related proteins (N = 416)				

## Data Availability

Details are presented within the paper in Section 3 and Supplementary Materials.

## Supplementary Materials

The following supporting information can be downloaded at: https://www.mdpi.com/article/10.3390/cells13110932/s1, Table S1: All proteins expressed in 7 days. Table S2: All proteins expressed in 14 days. Table S3: Osteogenesis and osteoclastogenesis-related proteins at 7 days. Table S4: Osteogenesis and osteoclastogenesis-related proteins at 14 days.

## References

[1] Petersen P.E., Baehni P.C. (2012). Periodontal health and global public health. Periodontology 2000.

[2] Wu L., Zhang S., Zhao L., Ren Z., Hu C. (2022). Global, regional, and national burden of periodontitis from 1990 to 2019: Results from the Global Burden of Disease study 2019. J. Periodontol..

[3] Chen L., Deng H., Cui H., Fang J., Zuo Z., Deng J., Li Y., Wang X., Zhao L. (2018). Inflammatory responses and inflammation-associated diseases in organs. Oncotarget.

[4] Algate K., Haynes D.R., Bartold P.M., Crotti T.N., Cantley M.D. (2016). The effects of tumour necrosis factor-α on bone cells involved in periodontal alveolar bone loss; osteoclasts, osteoblasts and osteocytes. J. Periodontal Res..

[5] AlZahrani S., Shinwari Z., Gaafar A., Alaiya A., Al-Kahtani A. (2022). Anti-Inflammatory Effect of Specialized Proresolving Lipid Mediators on Mesenchymal Stem Cells: An In Vitro Study. Cells.

[6] Narayanan L.L., Vaishnavi C. (2010). Endodontic microbiology. J. Conserv. Dent..

[7] Ali M., Yang F., Plachokova A.S., Jansen J.A., Walboomers X.F. (2021). Application of specialized pro-resolving mediators in periodontitis and peri-implantitis: A review. Eur. J. Oral Sci..

[8] Serhan C.N., Levy B.D. (2018). Resolvins in inflammation: Emergence of the pro-resolving superfamily of mediators. J. Clin. Investig..

[9] Serhan C.N., Chiang N., Dalli J. (2015). The resolution code of acute inflammation: Novel pro-resolving lipid mediators in resolution. Proceedings of the Seminars in Immunology.

[10] Serhan C.N. (2014). Pro-resolving lipid mediators are leads for resolution physiology. Nature.

[11] Arita M., Bianchini F., Aliberti J., Sher A., Chiang N., Hong S., Yang R., Petasis N.A., Serhan C.N. (2005). Stereochemical assignment, antiinflammatory properties, and receptor for the omega-3 lipid mediator resolvin E1. J. Exp. Med..

[12] Kantarci A., Aytan N., Palaska I., Stephens D., Crabtree L., Benincasa C., Jenkins B.G., Carreras I., Dedeoglu A. (2018). Combined administration of resolvin E1 and lipoxin A4 resolves inflammation in a murine model of Alzheimer’s disease. Exp. Neurol..

[13] Basil M.C., Levy B.D. (2016). Specialized pro-resolving mediators: Endogenous regulators of infection and inflammation. Nat. Rev. Immunol..

[14] Pan G., Zhang P., Yang J., Wu Y. (2022). The regulatory effect of specialized pro-resolving mediators on immune cells. Biomed. Pharmacother..

[15] Jordan P.M., Werz O. (2022). Specialized pro-resolving mediators: Biosynthesis and biological role in bacterial infections. FEBS J..

[16] Tsoyi K., Hall S.R.R., Dalli J., Colas R.A., Ghanta S., Ith B., Coronata A., Fredenburgh L.E., Baron R.M., Choi A.M.K. (2016). Carbon monoxide improves efficacy of mesenchymal stromal cells during sepsis by production of specialized proresolving lipid mediators. Crit. Care Med..

[17] Alghamdi F., Alhaddad A.J., Abuzinadah S. (2020). Healing of periapical lesions after surgical endodontic retreatment: A systematic review. Cureus.

[18] Ricucci D., Langeland K. (1998). Apical limit of root canal instrumentation and obturation, part 2. A histological study. Int. Endod. J..

[19] Azuma M.M., Gomes-Filho J.E., Ervolino E., Cardoso C.D.B.M., Pipa C.B., Kawai T., Conti L.C., Cintra L.T.A. (2018). Omega-3 fatty acids reduce inflammation in rat apical periodontitis. J. Endod..

[20] Azuma M.M., Gomes-Filho J.E., Ervolino E., Pipa C.B., Cardoso C.D.B.M., Andrada A.C., Kawai T., Cintra L.T.A. (2017). Omega 3 fatty acids reduce bone resorption while promoting bone generation in rat apical periodontitis. J. Endod..

[21] Colombo J.S., Moore A.N., Hartgerink J.D., D’Souza R.N. (2014). Scaffolds to control inflammation and facilitate dental pulp regeneration. J. Endod..

[22] Scarparo R.K., Dondoni L., Böttcher D.E., Grecca F.S., Figueiredo J.A.P., Kantarci A., Van Dyke T.E., Batista E.L. (2014). Intracanal delivery of Resolvin E1 controls inflammation in necrotic immature rat teeth. J. Endod..

[23] Wang C.-W., Yu S.H., Fretwurst T., Larsson L., Sugai J.V., Oh J., Lehner K., Jin Q., Giannobile W. (2020). V Maresin 1 promotes wound healing and socket bone regeneration for alveolar ridge preservation. J. Dent. Res..

[24] Yao D., Zou Y., Lv Y. (2022). Maresin 1 enhances osteogenic potential of mesenchymal stem cells by modulating macrophage peroxisome proliferator-activated receptor-γ-mediated inflammation resolution. Biomater. Adv..

[25] Albuquerque-Souza E., Schulte F., Chen T., Hardt M., Hasturk H., Van Dyke T.E., Holzhausen M., Kantarci A. (2020). Maresin-1 and resolvin E1 promote regenerative properties of periodontal ligament stem cells under inflammatory conditions. Front. Immunol..

[26] Jannaway M., Torrens C., Warner J.A., Sampson A.P. (2018). Resolvin E1, resolvin D1 and resolvin D2 inhibit constriction of rat thoracic aorta and human pulmonary artery induced by the thromboxane mimetic U46619. Br. J. Pharmacol..

[27] Chen J., Xu H., Xia K., Cheng S., Zhang Q. (2021). Resolvin E1 accelerates pulp repair by regulating inflammation and stimulating dentin regeneration in dental pulp stem cells. Stem Cell Res. Ther..

[28] Alkhayal Z., Shinwari Z., Gaafar A., Alaiya A. (2020). Proteomic profiling of the first human dental pulp mesenchymal stem/stromal cells from carbonic anhydrase II deficiency osteopetrosis patients. Int. J. Mol. Sci..

[29] Loi F., Córdova L.A., Pajarinen J., Lin T., Yao Z., Goodman S.B. (2016). Inflammation, fracture and bone repair. Bone.

[30] El Kholy K., Freire M., Chen T., Van Dyke T.E. (2018). Resolvin E1 promotes bone preservation under inflammatory conditions. Front. Immunol..

[31] Haworth O., Cernadas M., Yang R., Serhan C.N., Levy B.D. (2008). Resolvin E1 regulates interleukin 23, interferon-γ and lipoxin A4 to promote the resolution of allergic airway inflammation. Nat. Immunol..

[32] Souza D.G., Fagundes C.T., Amaral F.A., Cisalpino D., Sousa L.P., Vieira A.T., Pinho V., Nicoli J.R., Vieira L.Q., Fierro I.M. (2007). The required role of endogenously produced lipoxin A4 and annexin-1 for the production of IL-10 and inflammatory hyporesponsiveness in mice. J. Immunol..

[33] Yu N., Rakian A., Dean A., Van Dyke T.E. (2021). Specialized Proresolving Mediators Facilitate the Immunomodulation of the Periodontal Ligament Stem Cells. Front. Dent. Med..

[34] Dalli J., Zhu M., Vlasenko N.A., Deng B., Haeggström J.Z., Petasis N.A., Serhan C.N. (2013). The novel 13S, 14S-epoxy-maresin is converted by human macrophages to maresin 1 (MaR1), inhibits leukotriene A4 hydrolase (LTA4H), and shifts macrophage phenotype. FASEB J..

[35] Katarivas Levy G., Ong J., Birch M.A., Justin A.W., Markaki A.E. (2019). Albumin-enriched fibrin hydrogel embedded in active ferromagnetic networks improves osteoblast differentiation and vascular self-organisation. Polymers.

[36] Huang M., Wang Y., Wang Z., Qin Q., Zhang H., Liu S., Cui J., Zhang Y., Jiang X., Xu L. (2022). miR-134-5p inhibits osteoclastogenesis through a novel miR-134-5p/Itgb1/MAPK pathway. J. Biol. Chem..

[37] Fan T., Qu R., Jiang X., Yang Y., Sun B., Huang X., Zhou Z., Ouyang J., Zhong S., Dai J. (2021). Spatial organization and crosstalk of vimentin and actin stress fibers regulate the osteogenic differentiation of human adipose-derived stem cells. FASEB J..

[38] Zhou Y.K., Han C.S., Zhu Z.L., Chen P., Wang Y.M., Lin S., Chen L.J., Zhuang Z.M., Zhou Y.H., Yang R.L. (2024). M2 exosomes modified by hydrogen sulfide promoted bone regeneration by moesin mediated endocytosis. Bioact. Mater..

[39] Xie H., Cao T., Gomes J.V., Neto A.H.C., Rosa V. (2015). Two and three-dimensional graphene substrates to magnify osteogenic differentiation of periodontal ligament stem cells. Carbon.

[40] Kushwaha P., Alekos N.S., Kim S.P., Li Z., Wolfgang M.J., Riddle R.C. (2022). Mitochondrial fatty acid β-oxidation is important for normal osteoclast formation in growing female mice. Front. Physiol..

[41] Hu G., Yu Y., Sharma D., Pruett-Miller S.M., Ren Y., Zhang G.-F., Karner C.M. (2023). Glutathione limits RUNX2 oxidation and degradation to regulate bone formation. JCI Insight.

[42] Wen C., Zhou Y., Xu Y., Tan H., Pang C., Liu H., Liu K., Wei L., Luo H., Qin T. (2021). The regulatory role of GBF1 on osteoclast activation through EIF2a mediated ER stress and novel marker FAM129A induction. Front. Cell Dev. Biol..

[43] Feng Z., Su X., Wang T., Guo S. (2022). Identification of biomarkers that modulate osteogenic differentiation in mesenchymal stem cells related to inflammation and immunity: A bioinformatics-based comprehensive study. Pharmaceuticals.

[44] Marcon B.H., Rebelatto C.K., Cofré A.R., Dallagiovanna B., Correa A. (2020). DDX6 helicase behavior and protein partners in human adipose tissue-derived stem cells during early adipogenesis and osteogenesis. Int. J. Mol. Sci..

[45] Nantavisai S., Pisitkun T., Osathanon T., Pavasant P., Kalpravidh C., Dhitavat S., Makjaroen J., Sawangmake C. (2020). Systems biology analysis of osteogenic differentiation behavior by canine mesenchymal stem cells derived from bone marrow and dental pulp. Sci. Rep..

[46] Bai J., Zhang W., Zhou C., Zhao G., Zhong H., Hang K., Xu J., Zhang W., Chen E., Wu J. (2023). MFG-E8 promotes osteogenic differentiation of human bone marrow mesenchymal stem cells through GSK3β/β-catenin signaling pathway. FASEB J..

[47] Soysa N.S., Alles N. (2009). NF-κB functions in osteoclasts. Biochem. Biophys. Res. Commun..

[48] Zheng X., Tizzano M., Redding K., He J., Peng X., Jiang P., Xu X., Zhou X., Margolskee R.F. (2019). Gingival solitary chemosensory cells are immune sentinels for periodontitis. Nat. Commun..

[49] Czupalla C., Mansukoski H., Riedl T., Thiel D., Krause E., Hoflack B. (2006). Proteomic analysis of lysosomal acid hydrolases secreted by osteoclasts: Implications for lytic enzyme transport and bone metabolism. Mol. Cell. Proteom..

[50] Davis H.M., Valdez S., Gomez L., Malicky P., White F.A., Subler M.A., Windle J.J., Bidwell J.P., Bruzzaniti A., Plotkin L.I. (2019). High mobility group box 1 protein regulates osteoclastogenesis through direct actions on osteocytes and osteoclasts in vitro. J. Cell. Biochem..

[51] Yan B., Li Y., Min S., Zhang P., Xu B., Wang Z., Zhang W., Chen J., Luo G., Liu C. (2020). Effects of the bone/bone marrow microenvironments on prostate cancer cells and CD59 expression. Biomed. Res. Int..

[52] Chen F., Wang S., Zeng C., Tang S., Gu H., Wang Z., Li J., Feng P., Zhang Y., Wang P. (2023). Silencing circSERPINE2 restrains mesenchymal stem cell senescence via the YBX3/PCNA/p21 axis. Cell. Mol. Life Sci..

[53] Kim K., Punj V., Kim J.-M., Lee S., Ulmer T.S., Lu W., Rice J.C., An W. (2016). MMP-9 facilitates selective proteolysis of the histone H3 tail at genes necessary for proficient osteoclastogenesis. Genes Dev..

[54] Tadaki H., Saitsu H., Nishimura-Tadaki A., Imagawa T., Kikuchi M., Hara R., Kaneko U., Kishi T., Miyamae T., Miyake N. (2011). De novo 19q13. 42 duplications involving NLRP gene cluster in a patient with systemic-onset juvenile idiopathic arthritis. J. Hum. Genet..

[55] Huynh N., VonMoss L., Smith D., Rahman I., Felemban M.F., Zuo J., Rody W.J., McHugh K.P., Holliday L.S. (2016). Characterization of regulatory extracellular vesicles from osteoclasts. J. Dent. Res..

[56] Lu N.Z., Collins J.B., Grissom S.F., Cidlowski J.A. (2007). Selective regulation of bone cell apoptosis by translational isoforms of the glucocorticoid receptor. Mol. Cell. Biol..

[57] Handler D.C., Pascovici D., Mirzaei M., Gupta V., Salekdeh G.H., Haynes P.A. (2018). The Art of Validating Quantitative Proteomics Data. Proteomics.

[58] Mehta D., Ahkami A.H., Walley J., Xu S.L., Uhrig R.G. (2022). The incongruity of validating quantitative proteomics using western blots. Nat. Plants.

